# Therapeutic bronchoscopy in a lung abscess secondary to broncholithiasis

**DOI:** 10.1002/rcr2.487

**Published:** 2019-09-13

**Authors:** Yoshihiro Amano, Xuexia Tong, Kiyotaka Miura, Yukari Tsubata, Noriaki Kurimoto, Takeshi Isobe

**Affiliations:** ^1^ Department of Internal Medicine, Division of Medical Oncology & Respiratory Medicine Shimane University Faculty of Medicine Izumo Japan

**Keywords:** Broncholithectomy, broncholithiasis, lung abscess

## Abstract

A 60‐year‐old woman developed a pulmonary abscess due to broncholithiasis. It was treated by bronchoscopic broncholithectomy after antibiotic administration. Broncholithiasis is a rare disease and various treatment options are available, which include bronchoscopic broncholithectomy and surgery. Bronchoscopic broncholithectomy has proved to be more useful because it can be used to easily extract the broncholith by using a balloon and haemostasis can be obtained at the same time.

## Introduction

Broncholithiasis is a condition in which calcified material (a broncholith) is present within a bronchus or a cavity communicating with a bronchus 1. It is relatively uncommon; in Japan, broncholithiasis account for 0.1–0.2% of respiratory diseases. A broncholith typically causes clinical problems by the erosion and extrusion of calcification from an adjacent lymph node into the bronchial lumen 2. The most common cause in Japan is infection with *Mycobacterium tuberculosis* 2. Other causes include infection with *Histoplasma capsulatum*, or cryptococcosis, coccidioidomycosis, actinomycosis, aspergillosis, nocardiosis, silicosis, and/or malignancy. Pulmonary signs and symptoms are non‐specific 3.

## Case Report

**Figure 1:**
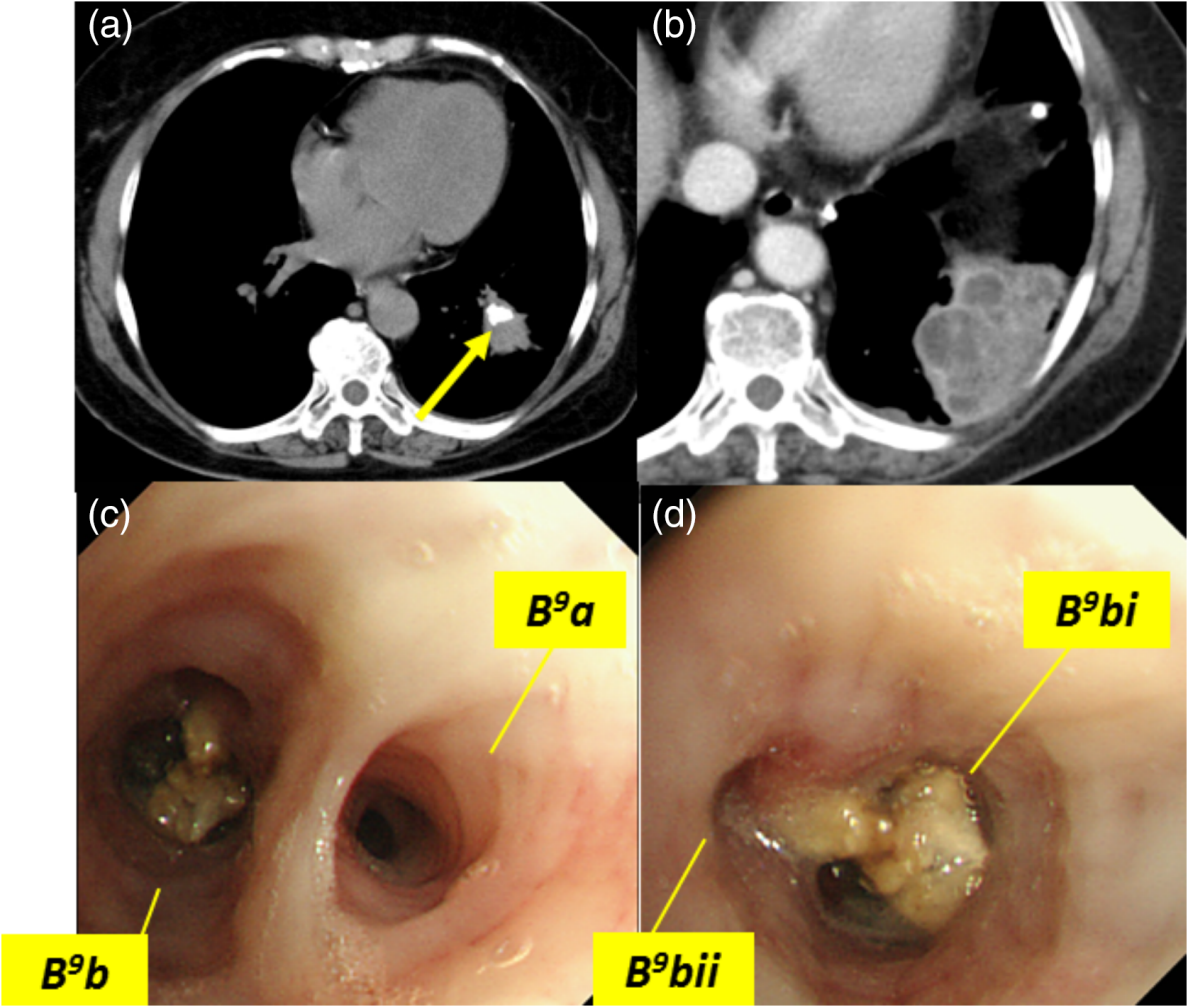
(A) Chest computed tomography (CT) showing calcified nodules distributed in the left lower lobe. (B) Chest CT showing calcified nodules in the left lower lobe combined with consolidation. (C) Bronchoscopic examination showing left lower lobe and basal segmental bronchial abscess.

A 60‐year‐old woman presented to the hospital with persistent cough, left‐side back pain, and fever since the past one month. Chest radiography revealed left lower lobe infiltrate. A provisional diagnosis of pneumonia was made and antibiotics were prescribed for a week with no improvement. She was then admitted to our hospital because of the same complaint of persistent cough. Her medical history revealed hypertension. She did not have any past history of tuberculosis and interferon‐gamma release assay was negative. Chest computed tomography (CT) showed calcified nodules distributed in the mediastinum and a single calcified nodule with consolidation in the left lower lobe (Fig. 1A, B). The left lower lobe nodule was combined with consolidation (Fig. 1B). After the patient's course of antibiotics was extended for one week, re‐examination through chest CT revealed that the consolidation was reduced but was still evident. The patient's condition improved and she was discharged. Eleven days later, re‐examination through chest CT showed that left lower lobe consolidation was still evident without much change. Bronchoscopic examination revealed purulent material in the left lower lobe common to basal stem bronchus (Fig. 1C). Subsequently, bronchoscopic biopsy was performed. Culture of the bronchoscopic specimen did not reveal anything in particular, including tuberculous or non‐tuberculous mycobacteria. A pathological diagnosis of broncholithiasis was made.

**Figure 2:**
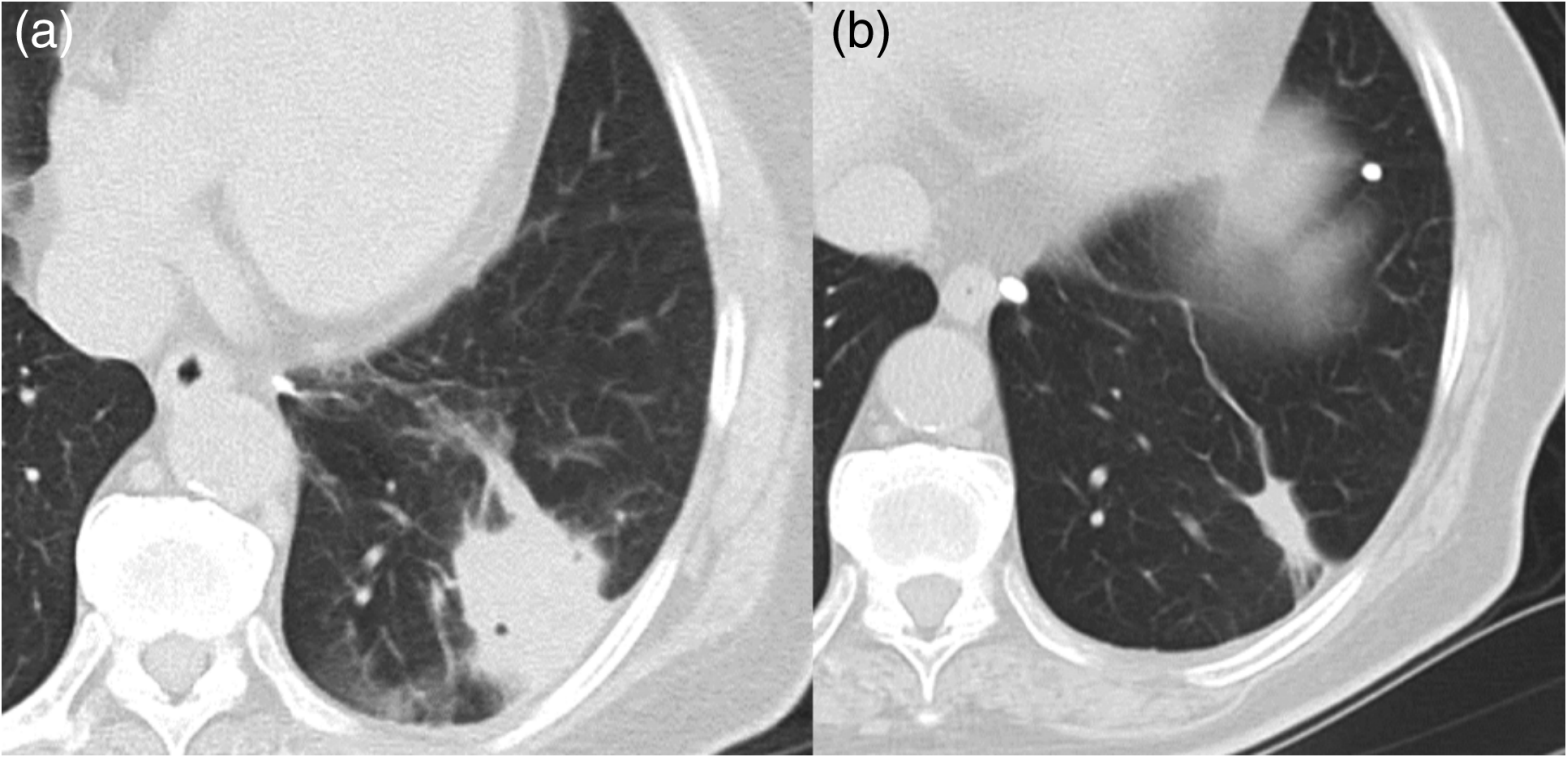
Chest computed tomography (CT) revealed consolidation before the removal of the broncholiths (A), and three months later, CT showed gradual regression of consolidation (B).

Two weeks later, the patient again underwent a bronchoscopic examination, she was intubated, and we used the technique of aspiration using a bronchoscope to suck all the abscess tissue. Flexible bronchoscopy forceps were used to remove the broncholiths, which proved to be quite difficult. As the broncholiths were completely in the lumen of the airway and mobile, we shifted to a balloon catheter to extract all the broncholiths. A disposable balloon catheter (B5‐2C®; Olympus, Japan) was inserted beside and beyond the intra‐luminal broncholiths, the balloon inflated, and the broncholiths were removed by withdrawing the bronchoscope and catheter with the balloon inflated. Three months after broncholith removal, the patient was re‐examined with serial chest CT scans, which revealed that the left lower lobe consolidation and calcification gradually regressed and became normal (Fig. 2).

## Discussion

This case report describes a useful technique of bronchoscopy using a balloon catheter for treating bronchial abscess due to broncholithiasis. This is a relatively unconventional method of removal of broncholiths, especially in cases where bronchoscopic forceps would be too difficult to use due to the fragile nature of the broncholiths. It can open new arenas in broncholith removal by avoiding surgery or bleeding complications.

Various treatment options are available, which include bronchoscopic broncholithectomy and surgery. The indications for surgery include the following: recurrent haemoptysis, pulmonary abscess, and cancer. Similar cases of successful endoscopic removal of a broncholith not fixed in the airway or partially mobile on bronchoscopic probing, and small enough to be removed endoscopically have been reported; however, it is often not the first choice of treatment.

In our case, we decided that before bronchoscopic removal the operator should confirm that (1) broncholiths are present in the bronchial lumen completely, not prone to bleeding, which might be the case if granulation tissue is present on the surface of broncholiths, and enough space is evident for securing bronchoscopy forceps; (2) broncholiths are loose and mobile, as too firm broncholiths increase the risk of bleeding while removal; and (3) radiography and CT scans reveal no enlarged bronchial artery or collateral vessels around the broncholiths.

During bronchoscopic removal, it was difficult to handle the forceps as broncholiths are smooth, hard, and fragile. In such cases, we suggest the use of a balloon catheter to extract the broncholiths. Moreover, the use of a balloon catheter provides a strong haemostatic effect during removal. A previous report indicated that flexible bronchoscopy extraction using balloons and forceps for free broncholiths had a 100% success rate, and for penetrating broncholiths had a 30% success rate 4. Caution must be exercised in the safe removal of broncholiths, as an unexpected respiratory tract haemorrhage may occur during the procedure. Hence, it is always advisable to use oral intubation during bronchoscopic removal.

In conclusion, we presented the use of a bronchoscopic intervention in case of lung abscess due to broncholithiasis. We concluded that if the broncholiths were exposed to the lumen of the bronchus completely, and no granulation tissue hyperplasia or enlarged vessels were evident around it, bronchoscopic removal should be the treatment of choice and surgery could be avoided. If bronchoscopic forceps are difficult to handle, we recommend the use of a balloon catheter to extract the broncholith as well as to get a haemostatic effect.

### Disclosure Statement

Appropriate written informed consent was obtained for publication of this case report and accompanying images.
